# A web-based prediction model for overall survival of elderly patients with early renal cell carcinoma: a population-based study

**DOI:** 10.1186/s12967-022-03287-w

**Published:** 2022-02-14

**Authors:** Jinkui Wang, Jie Tang, Tiaoyao Chen, Song Yue, Wanting Fu, Zulong Xie, Xiaozhu Liu

**Affiliations:** 1 Department of Cardiology, The Second Affiliated Hospital of Chongqing Medical University, Chongqing, China; 2 Department of Urology, Chongqing Key Laboratory of Children Urogenital Development and Tissue Engineering, Chongqing Key Laboratory of Pediatrics, Ministry of Education Key Laboratory of Child Development and Disorders, National Clinical Research Center for Child Health and Disorders, China International Science and Technology Cooperation base of Child development and Critical Disorders, Children’s Hospital of Chongqing Medical University, Chongqing, China; 3 Department of Biostatistics and Epidemiology, Public Health School, Shenyang Medical College, Shenyang, China; 4 College of Medical Informatics, The Chongqing Medical University, Chongqing, China; 5 Department of Gynecology and Obstetrics, The Second Affiliated Hospital of Chongqing Medical University, Chongqing, China; 6 College of Traditional Chinese Medicine, Guangzhou University of Chinese Medicine, Guangzhou, China

**Keywords:** Nomogram, Elderly patients, Early RCC, Overall survival, SEER, Online application

## Abstract

**Background:**

The number of elderly patients with early renal cell carcinoma (RCC) is on the rise. However, there is still a lack of accurate prediction models for the prognosis of early RCC in elderly patients. It is necessary to establish a new nomogram to predict the prognosis of elderly patients with early RCC.

**Methods:**

The data of patients aged above 65 years old with TNM stage I and II RCC were downloaded from the SEER database between 2010 and 2018. The patients from 2010 to 2017 were randomly assigned to the training cohort (n = 7233) and validation cohort (n = 3024). Patient data in 2018(n = 1360) was used for external validation. We used univariable and multivariable Cox regression model to evaluate independent prognostic factors and constructed a nomogram to predict the 1-, 3-, and 5-year overall survival (OS) rates of patients with early-stage RCC. Multiple parameters were used to validate the nomogram, including the consistency index (C-index), the calibration plots, the area under the receiver operator characteristics (ROC) curve, and the decision curve analysis (DCA).

**Results:**

The study included a total of 11,617 elderly patients with early RCC. univariable and multivariable Cox regression analysis based on predictive variables such as age, sex, histologic type, Fuhrman grade, T stage, surgery type, tumors number, tumor size, and marriage were included to establish a nomogram. The C-index of the training cohort and validation cohort were 0.748 (95% CI: 0.760–0.736) and 0.744 (95% CI: 0.762–0.726), respectively. In the external validation cohort, C-index was 0.893 (95% CI: 0.928–0.858). The calibration plots basically coincides with the diagonal, indicating that the observed OS was almost equal to the predicted OS. It was shown in DCA that the nomogram has more important clinical significance than the traditional TNM stage.

**Conclusion:**

A novel nomogram was developed to assess the prognosis of an elderly patient with early RCC and to predict prognosis and formulate treatment and follow-up strategies.

## Introduction

Among all malignant tumors, kidney cancer is one of the most common tumors in the world, accounting for 3.7% of global cancers, and the incidence of kidney cancer is gradually increasing [1–4]. Renal cell carcinoma (RCC) is a malignant tumor derived from renal tubular cells. It is the most common histopathological subtype of renal cancer, accounting for approximately 85% of renal cancers [2, 3]. Clear cell RCC is the most common type of RCC, accounting for 82–90% [5]. RCC is more common in men with a male to female incidence ratio of 1.7:1 [3, 6]. Some authors have investigated whether kidney cancer manifests differently in young and elderly patients. The results show that the cancer-specific survival rate of young patients is higher than that of elderly patients [7–10]. In recent years, the number of patients with small renal masses has increase and compared with the young patients, the elderly (i.e., 65 years and older) patients have a higher diagnosis rate [11, 12]. Although the treatment of kidney cancer has been continuously improved, the mortality rate of elderly kidney cancer patients still remained high [13, 14]. Therefore, accurate prognostic prediction for elderly patients with early RCC is necessary, which may help clinicians make better decisions.

The TNM staging system is the standard method for most clinicians and medical researchers to classify malignant tumors and is widely used in cancer treatment evaluation and prognosis evaluation [15]. However, the TNM system does not include clinicopathological factors that may have an important impact on the prognosis of RCC, such as age, sex, surgical methods, tumor grade, and lymphadenectomy [15, 16]. There is still a lack of survival prediction models for elderly patients with early RCC so that the need for constructing a reliable and accurate prognostic model is necessary.

A nomogram is a user-friendly graphical mathematical model that predicts the occurrence of a given event by generating a single numerical estimate based on specific clinical and pathological variables [17–19]. As a visual scoring graph, the establishment of the nomogram is theoretically based on the traditional Cox proportional risk regression model, and the establishment of the nomogram does not sacrifice the accuracy of the regression model. With the predictive power of conventional regression models and the excellent performance of being user friendly and easy to use, we used nomograms to predict patient survival.

In previous studies, the nomogram model was developed to improve clinical decision-making, such as liver cancer, lung cancer, breast cancer [20–22]. So far, the nomogram model for predicting the prognosis of elderly patients with early RCC has not been established using the Surveillance Epidemiology and End Results (SEER) database. Herein, we designed a nomogram combined with the clinicopathological parameters extracted from the SEER database to predict the prognosis of early RCC elderly patients, which may have potential clinical application value.

## Patients and methods

### Data source and data extraction

The original clinical data was extracted from the National Cancer Institute's Surveillance, Epidemiology, and End Results (SEER) Program to identify patients older than 65 years old and diagnosed with TNM stage I and II RCC in the United States from 2010 to 2018. The data analyzed in this study is available on the SEER database (http://seer.cancer.gov/), which covers approximately 28% of Americans and contains 18 population-based tumor registries [23]. The patient's demographic information, tumor characteristics and survival status are all publicly available through the SEER database. Because we use publicly anonymous data, our research does not require ethical review or patient consent. Our research method complies with the rules and regulations of the SEER database.

**Fig. 1:**
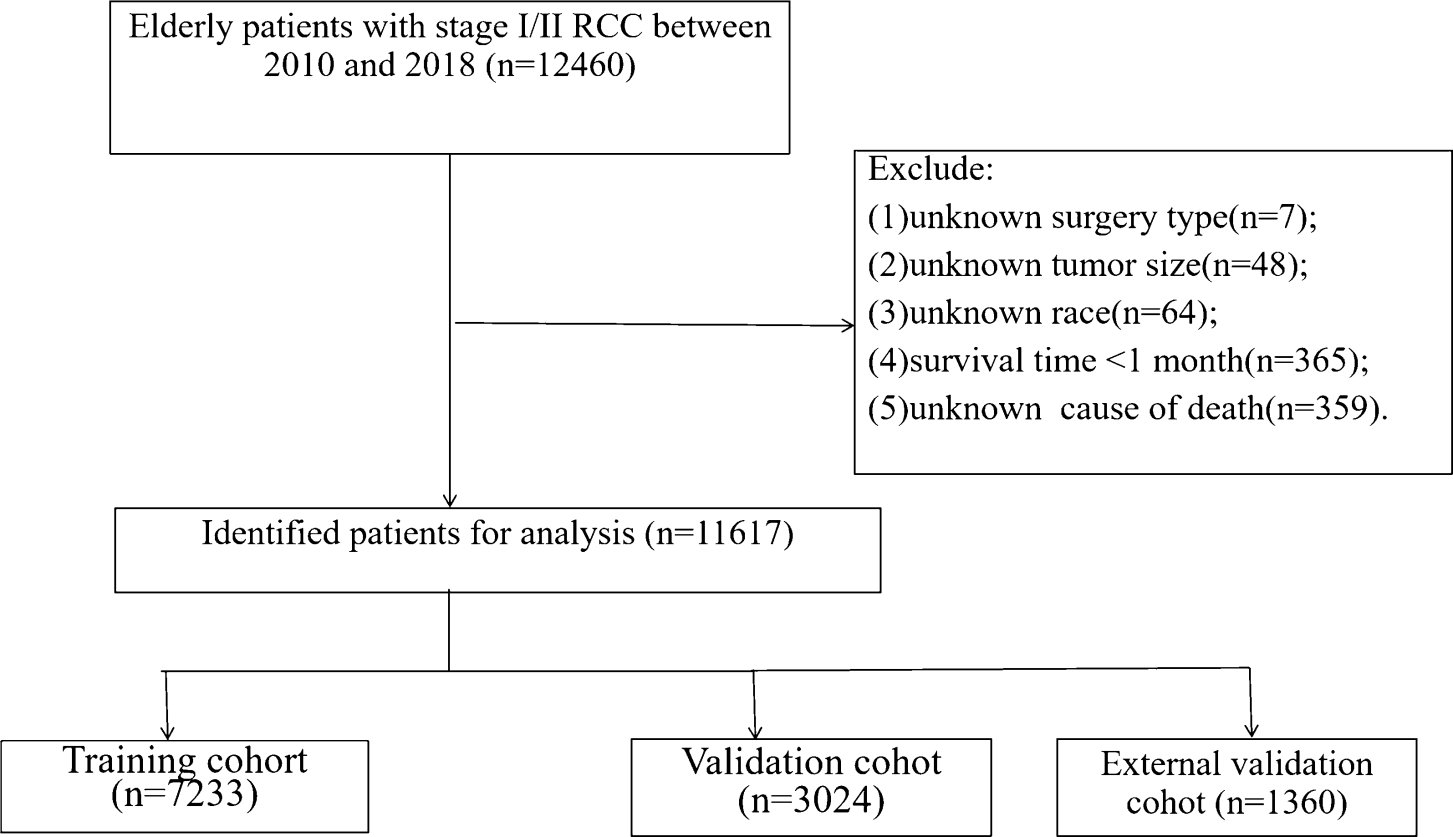
The flowchart of including and dividing patients

Excluding unknown or missing clinicopathological information, a total of 11,617 patients were included in this study. Demographic and clinical data include age at diagnosis, sex, race, tumor laterality, TNM stage, histological type, Fuhrman grade, type of surgery, chemotherapy, radiotherapy, life status, survival time, and marital status were collected. The selection criteria are: (1) age ≥ 65 years; (2) pathological diagnosis of renal cell carcinoma; (3) T1/T2, N0, M0; (4) Unilateral kidney cancer. The exclusion criteria are: (1) unknown surgery type; (2) unknown tumor size; (3) unknown race; (4) survival time < 1 month. The flowchart for selecting patients is shown in Fig. 1.

The patient's race includes white, black, and other races (American Indian/AK Native, Asian/Pacific Islander). Fuhrman grades I, II, III, and IV represent well-differentiated, moderately-differentiated, poorly-differentiated, and undifferentiated, respectively. The surgical methods are divided into four groups according to the SEER Kidney Surgery Codes 2018: non-surgical group (code 0), local tumor excision (code 10–27; includes cryosurgery, thermal ablation, laser excision), and partial nephrectomy (PN, code 30) and radical nephrectomy (RN, code 40–80).

### Statistical analysis

We randomly subdivided 10,595 patients from 2010 to 2017 into a training cohort 70%(n = 7233) and a validation cohort 30%(n = 3024). Patients in 2018(n = 1360) were used for external validation. The Cox proportional-hazards risk model was used for univariable and multivariable analysis to evaluate independent risk factors for RCC, the hazard ratio (HR) and 95% confidence interval (CI) were recorded. Important parameters were identified in the multivariable analysis and included in the nomogram for predicting the prognosis of RCC of elderly patients. A new nomogram model was established to estimate the OS rates of elderly patients with RCC in 1-year, 3-year and 5-year. The predictive model is essentially the visualization of multivariable Cox regression. When constructing the multivariate Cox regression, we obtained the regression coefficient β (coef) of each variable; we can also get a specific score for each variable in the constructed Nomogram. Nomogram standardizes the regression coefficients and displays them as risk scores on a number line.

The predictive reliability and accuracy of the nomograph were evaluated by the SEER internal validation cohort. We constructed the receiver operating characteristic (ROC) curve to obtain the area under the curve (AUC) and a corrected C-Index to reflect the discriminability and predictive accuracy of the nomogram. We constructed 1000 bootstrap resamples and used the calibration plots to validate the nomogram internally. At the same time, we performed analysis on patients' cancer-specific survival (CSS).

Decision curve analysis (DCA) was used to assess the clinical significance of our nomogram [24]. It is a new algorithm for evaluating the clinical utility value of nomogram by estimating the net benefit under each risk threshold. Patients were divided into low-risk, medium-risk, and high-risk groups according to nomogram total cut-off values. Kaplan–Meier curve and log-rank test were used to compare the survival rates of patients between different groups.

All statistical analyses and charts were performed using R software version 4.1.0 and SPSS version 26.0. We used the "RMS", "DynNom", "survival" and "ggDCA" R packages to construct and validate the nomogram, formulate the ROC curve, and establish DCA. When the P value is less than 0.05, the result is considered statistically significant (two-side).

## Results

### Clinical features

We included 11,617 elderly RCC patients from the SEER database who met our study criteria according to our inclusion and exclusion criteria. Among them, patients from 2010 to 2017(n = 10,257) were used for the establishment and internal validation of predictive models. The clinicopathologic features of RCC in elderly patients in the training and validation cohorts are shown in Table 1. Among these patients, 6406 (62.5%) patients were males, 8300 (80.9%) patients were the white race, 6231 (60.7%) patients were married, and 6309 (61.5%) patients were stage T1a. 993 (9.68%), 3923 (38.2%), 1846 (18.0%), and 177 (1.73%) patients whose Fuhrman grade were I, II, III, and IV, respectively. 1446 (14.1%), 2973 (29.0%), and 3798 (37.0%) patients who had undergone local tumor excision, partial nephrectomy (PN), and radical nephrectomy (RN), respectively. There were no significant differences in clinicopathologic characteristics between the training and the validation cohort.

**Table 1:** Clinicopathological characteristics of patients with RCC

	Total N = 10,595	Training cohort N = 7233	Validation cohort N = 3024	p
Age	73.5 (6.73)	73.5 (6.69)	73.5 (6.83)	0.7
Race				0.4
White	8300 (80.9%)	5847 (80.8%)	2453 (81.1%)	
Black	1132 (11.0%)	815 (11.3%)	317 (10.5%)	
Other^a^	825 (8.04%)	571 (7.89%)	254 (8.40%)	
Sex				0.4
Male	6406 (62.5%)	4498 (62.2%)	1908 (63.1%)	
Female	3851 (37.5%)	2735 (37.8%)	1116 (36.9%)	
Marriage				0.5
No	4026 (39.3%)	2856 (39.5%)	1170 (38.7%)	
Married	6231 (60.7%)	4377 (60.5%)	1854 (61.3%)	
Year of diagnosis				0.06
2010–2013	4815 (46.9%)	3352 (46.3%)	1463 (48.4%)	
2014–2017	5442 (53.1%)	3881 (53.7%)	1561 (51.6%)	
Histologictype				0.5
Clear cell	5539 (54.0%)	3932 (54.4%)	1607 (53.1%)	
Papillary	1658 (16.2%)	1173 (16.2%)	485 (16.0%)	
Chromophobe	578 (5.64%)	407 (5.63%)	171 (5.65%)	
Other^b^	2482 (24.2%)	1721 (23.8%)	761 (25.2%)	
Laterality				1
Left	4996 (48.7%)	3523 (48.7%)	1473 (48.7%)	
Right	5261 (51.3%)	3710 (51.3%)	1551 (51.3%)	
T stage				0.9
T1a	6309 (61.5%)	4452 (61.6%)	1857 (61.4%)	
T1b	2907 (28.3%)	2045 (28.3%)	862 (28.5%)	
T2a	764 (7.45%)	538 (7.44%)	226 (7.47%)	
T2b	277 (2.70%)	198 (2.74%)	79 (2.61%)	
Grade				0.7
I	993 (9.68%)	715 (9.89%)	278 (9.19%)	
II	3923 (38.2%)	2782 (38.5%)	1141 (37.7%)	
III	1846 (18.0%)	1293 (17.9%)	553 (18.3%)	
IV	177 (1.73%)	123 (1.70%)	54 (1.79%)	
Unknown	3318 (32.3%)	2320 (32.1%)	998 (33.0%)	
Surgery				0.044
No	2040 (19.9%)	1387 (19.2%)	653 (21.6%)	
Local excision^c^	1446 (14.1%)	1035 (14.3%)	411 (13.6%)	
Partial Nephrectomy	2973 (29.0%)	2119 (29.3%)	854 (28.2%)	
Radical Nephrectomy	3798 (37.0%)	2692 (37.2%)	1106 (36.6%)	
Chemotherapy				0.9
No/unknown	10,182 (99.3%)	7179 (99.3%)	3003 (99.3%)	
Yes	75 (0.73%)	54 (0.75%)	21 (0.69%)	
Radiation				0.9
No/unknown	10,223 (99.7%)	7210 (99.7%)	3013 (99.6%)	
Yes	34 (0.33%)	23 (0.32%)	11 (0.36%)	
Total number of tumors	1.62 (0.84)	1.62 (0.84)	1.61 (0.82)	0.7
Tumor size	41.1 (27.2)	41.1 (26.9)	41.0 (28.1)	0.9
Survival months	47.4 (28.1)	47.3 (28.2)	47.8 (27.9)	0.4
Cancer-specific survival				0.8
Dead	786 (7.66%)	558 (7.71%)	228 (7.54%)	
Alive	9471 (92.3%)	6675 (92.3%)	2796 (92.5%)	
Overall survival				0.5
Dead	2623 (25.6%)	1836 (25.4%)	787 (26.0%)	
Alive	7634 (74.4%)	5397 (74.6%)	2237 (74.0%)	

^a^Other includes Asian/Pacific Islander, American Indian/Alaskan Native;

^b^Others includes the pathological type of RCC is not known

^c^Local excision includes cryosurgery, thermal ablation, laser excision

### Univariable and multivariable Cox regression analysis

We used univariable regression to identify eight significant risk factors, including age, sex, race, histologic type, Fuhrman grade, T stage, surgery type, tumors number, tumor size, and marriage (Table 2). Next, we used the selected factors to establish a multivariable Cox model to determine independent risk factors. The hazard ratio (HR) is presented to quantify its impact on OS. The results of multivariable analysis of the training cohort are shown in Table 2. Variables including age, sex, race, histologic type, Fuhrman grade, T stage, surgery type, tumors number, tumor size, and marriage. In general, ten parameters are considered to be significant independent risk factors and they may effectively predict OS in elderly RCC patients. At the same time, univariable and multivariable analysis suggested that age, histologic type, Fuhrman grade, T stage, surgery type, radiotherapy, and tumor number were independent risk factors for CSS in patients (Table 3).

**Table 2:** Univariable and multivariable Cox regression analysis of OS in training cohort

	Univariate			Multivariable		
	HR	95%CI	P	HR	95%CI	P
Age	1.09	1.08–1.09	< 0.001	1.048	1.041–1.056	< 0.001
Race						
White	Reference			Reference		
Black	1.1	0.95–1.26	0.2	1.168	1.011–1.35	0.035
Other^a^	0.82	0.68–0.98	0.031	0.919	0.763–1.107	0.4
Sex						
Male	Reference			Reference		
Female	0.9	0.81–0.99	0.023	0.783	0.707–0.868	< 0.001
Year of diagnosis						
2010–2013	Reference					
2014–2017	1.01	0.91–1.12	0.9			
Histologic type						
Clear cell	Reference			Reference		
Papillary	0.91	0.78–1.06	0.2	0.837	0.717–0.975	0.023
Chromophobe	0.74	0.57–0.95	0.017	0.678	0.525–0.876	0.003
Other^b^	2.59	2.34–2.86	< 0.001	1.122	0.979–1.287	0.1
Laterality						
Left	Reference					
Right	1.01	0.92–1.11	0.9			
T stage	1.22	1.15–1.29	< 0.001	1.23	1.134–1.335	< 0.001
Grade						
I	Reference			Reference		
II	1.04	0.86–1.26	0.7	1.132	0.935–1.371	0.2
III	1.17	0.95–1.43	0.14	1.279	1.034–1.584	0.024
IV	2.34	1.68–3.27	< 0.001	2.241	1.598–3.143	< 0.001
Unknown	2.6	2.17–3.12	< 0.001	1.097	0.895–1.344	0.375
Surgery						
No	Reference			Reference		
Local excision^c^	0.28	0.24–0.33	< 0.001	0.408	0.348–0.48	< 0.001
Partial Nephrectomy	0.14	0.12–0.16	< 0.001	0.227	0.189–0.272	< 0.001
Radical Nephrectomy	0.25	0.22–0.28	< 0.001	0.311	0.264–0.366	< 0.001
Radiotherapy						
No/unknown	Reference					
Yes	3.16	1.86–5.34	< 0.001			
Chemotherapy						
No/Unknown	Reference					
Yes	3.48	2.46–4.94	< 0.001			
Tumors number	1.27	1.21–1.33	< 0.001	1.192	1.136–1.249	< 0.001
Marriage						
No	Reference			Reference		
Married	0.67	0.61–0.73	< 0.001	0.807	0.731–0.891	< 0.001
Tumor size	1.001	1.001–1.002	< 0.001	1.002	1.001–1.004	0.003

^a^Other includes Asian/Pacific Islander, American Indian/Alaskan Native;

^b^Others includes the pathological type of RCC is not known

^c^Local excision includes cryosurgery, thermal ablation, laser excision

**Table 3:** Univariable and multivariable Cox regression analysis of CSS in training cohort

	Univariate			Multivariable		
	HR	95%CI	P	HR	95%CI	P
Age	1.09	1.08–1.1	< 0.001	1.05	1.038–1.063	< 0.001
Race						
White	Reference					
Black	0.96	0.73–1.26	0.8			
Other^a^	0.93	0.68–1.27	0.6			
Sex						
Male	Reference					
Female	1	0.84–1.18	0.9			
Year of diagnosis						
2010–2013	Reference					
2014–2017	0.98	0.81–1.18	0.8			
Histologic type						
Clear cell	Reference			Reference		
Papillary	0.89	0.68–1.15	0.4	0.852	0.65–1.117	0.2
Chromophobe	0.6	0.36–0.97	0.038	0.517	0.314–0.851	0.009
Other^b^	2.28	1.9–2.73	< 0.001	0.921	0.717–1.184	0.5
Laterality						
Left	Reference					
Right	1.13	0.96–1.34	0.15			
T stage	1.69	1.55–1.84	< 0.001	1.831	1.661–2.018	< 0.001
Grade						
I	Reference			Reference		
II	1.62	1.07–2.45	0.022	1.744	1.148–2.65	0.009
III	2.07	1.34–3.19	0.001	2.155	1.379–3.368	0.001
IV	5.64	3.2–9.96	< 0.001	4.397	2.462–7.852	< 0.001
Unknown	3.99	2.66–5.97	< 0.001	1.777	1.15–2.746	0.01
Surgery						
No	Reference			Reference		
Local excision^c^	0.25	0.19–0.33	< 0.001	0.403	0.297–0.547	< 0.001
Partial Nephrectomy	0.11	0.08–0.15	< 0.001	0.166	0.117–0.236	< 0.001
Radical Nephrectomy	0.3	0.25–0.36	< 0.001	0.26	0.194–0.349	< 0.001
Radiotherapy						
No/unknown	Reference			Reference		
Yes	5.9	2.94–11.87	< 0.001	2.554	1.254–5.2	0.01
Chemotherapy						
No/unknown	Reference					
Yes	5.29	3.16–8.84	< 0.001	Reference		
Tumors number	1.2	1.11–1.31	< 0.001	1.166	1.068–1.273	0.001
Marriage						
No	Reference					
Married	0.67	0.56–0.79	< 0.001			
Tumor size	1.01	1–1.01	< 0.001			

^a^Other includes Asian/Pacific Islander, American Indian/Alaskan Native

^b^Others includes the pathological type of RCC is not known

^c^Local excision includes cryosurgery, thermal ablation, laser excision

### Nomogram construction for 1-year, 3-year, and 5-year OS and CSS

**Fig. 2:**
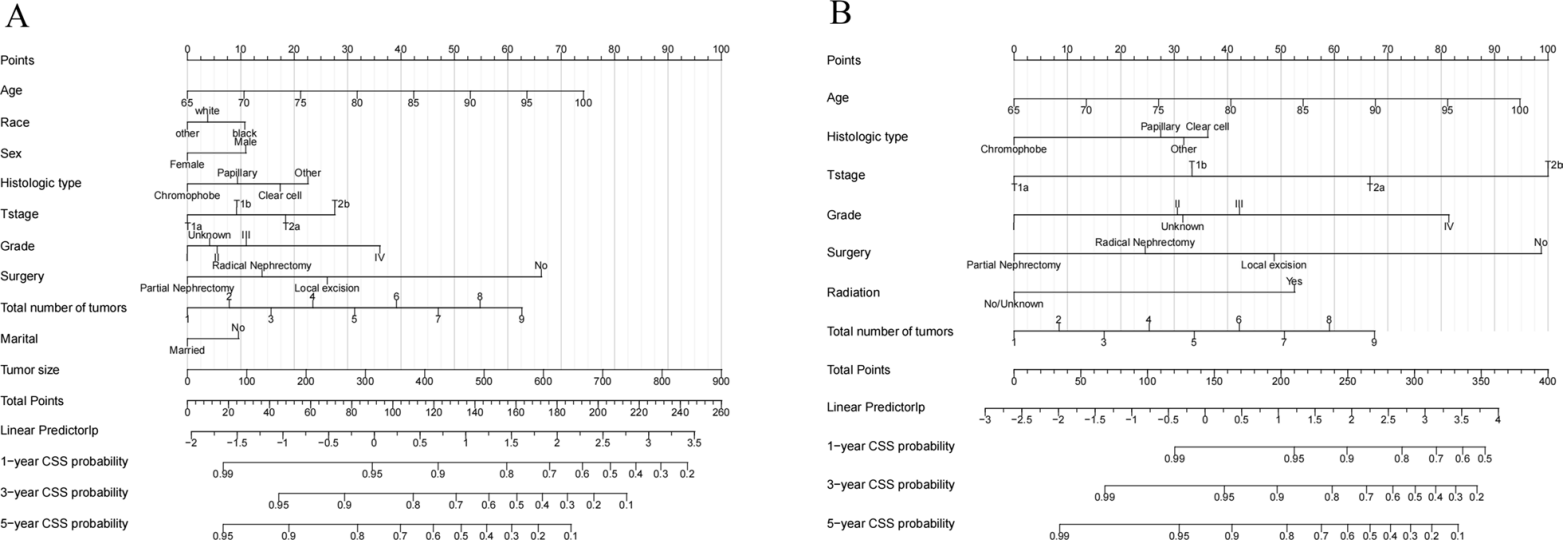
Nomograms for 1-, 3-, and 5-year OS and CSS of patients with RCC

We constructed a nomogram model using the eight significant risk factors identified by multivariable Cox regression analysis of the training cohort and listed the corresponding score for each parameter (Fig. 2A). The nomogram can estimate the 1 -, 3 -, and 5-year OS of the training cohort. As we can see from the nomogram, surgery and age had the greatest impact on OS, followed by tumor size, Fuhrman grade, histological type, tumor number, T stage and marriage. Meanwhile, we established a nomogram to predict patients' CSS (Fig. 2B).

### Validation of the nomogram

**Fig. 3:**
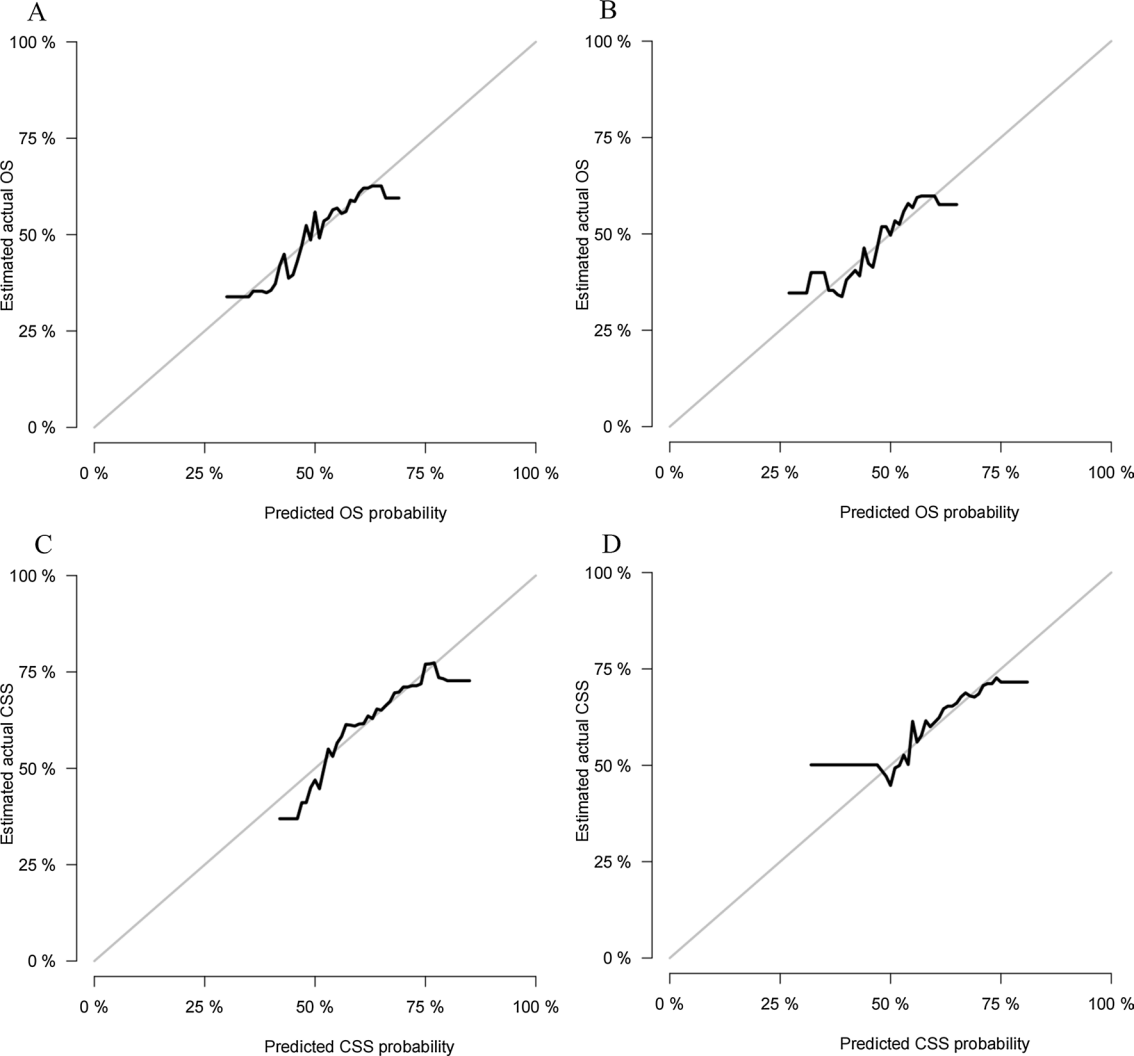
Calibration curves of nomogram. **A** For OS in training cohort; **B** For OS in validation cohort; **C** For CSS in training cohort; **D** For CSS in validation cohort

**Fig. 4:**
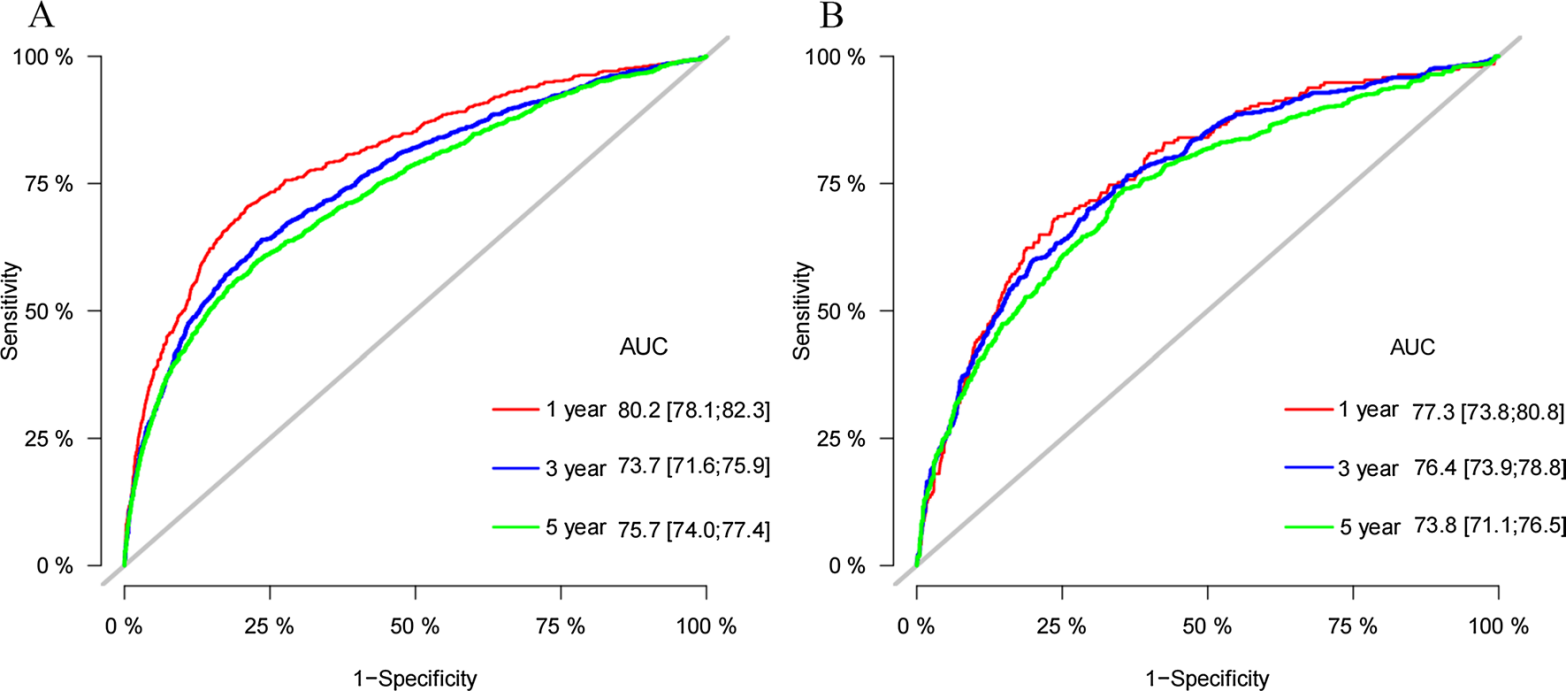
The ROC for OS of 1-, 3- and 5-year of training cohort (**A**) and validation cohort (**B**)

The C-index of the training cohort and validation cohort were 0.748 (95% CI: 0.760–0.736) and 0.744 (95% CI: 0.762–0.726), respectively, indicating that the model had good discriminatory power. In the external validation cohort, C-index was 0.893 (95% CI: 0.928–0.858). The calibration plots for the training cohort and the validation cohort used to predict OS show good agreement between the actual observations and the model predictions (Fig. 3A, B). The calibration plots of the nomogram for predicting patients' CSS still showed good accuracy (Fig. 3C, D). In the training cohort, the AUC of predicted nomogram for 1-year, 3-year and 5-year were 0.802, 0.737 and 0.757(Fig. 4A). In the validation cohort, the AUC of predicted nomogram for 1-year, 3-year and 5-year were 0.773, 0.764 and 0.738(Fig. 4B).

### Clinical application of the nomogram

**Fig. 5:**
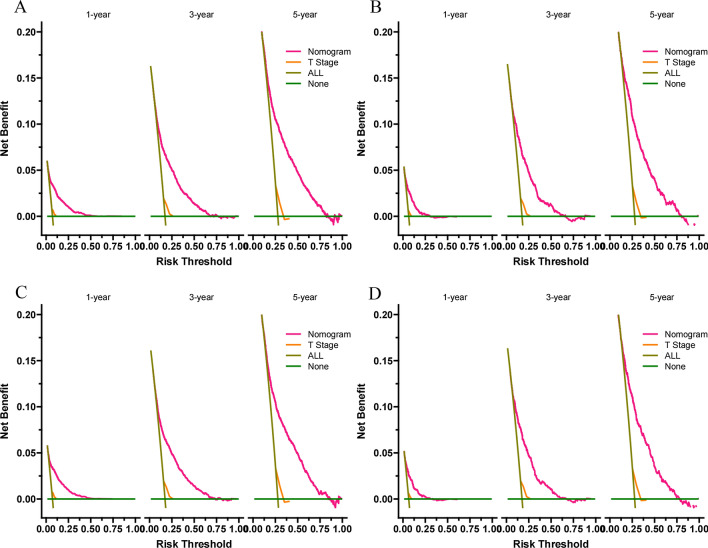
Decision curves of the nomogram predicting OS in training cohort (**A**) and validation cohort (**B**). Decision curves of the nomogram predicting CSS in training cohort (**C**) and validation cohort (**D**). The y-axis represents the net benefit, and the x-axis represents the threshold probability. The purple line indicates that no patients have died, and the blue line indicates that all patients have died. When the threshold probability is between 20 and 60%, the net benefit of the model exceeds all deaths or no deaths

**Fig. 6:**
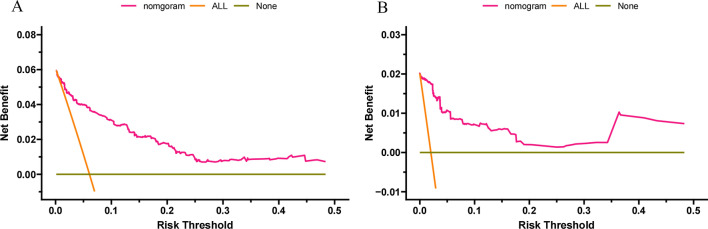
Decision curves of the nomogram predicting OS in external validation cohort (**A**), the nomogram predicting CSS in external validation cohort (**B**)

**Fig. 7:**
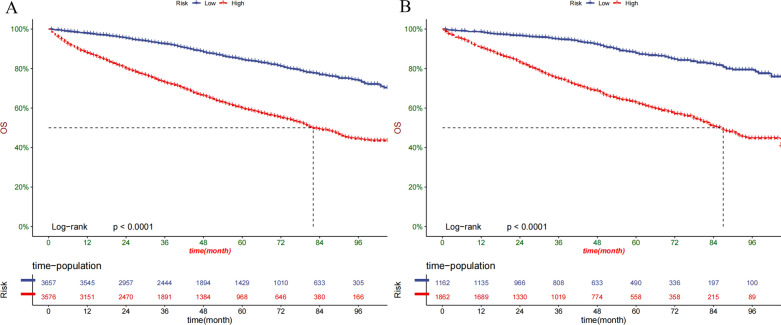
Kaplan–Meier curves of OS for patients in the low-, and high-risk groups in the training Cohort (**A**) and validation Cohort (**B**)

**Fig. 8:**
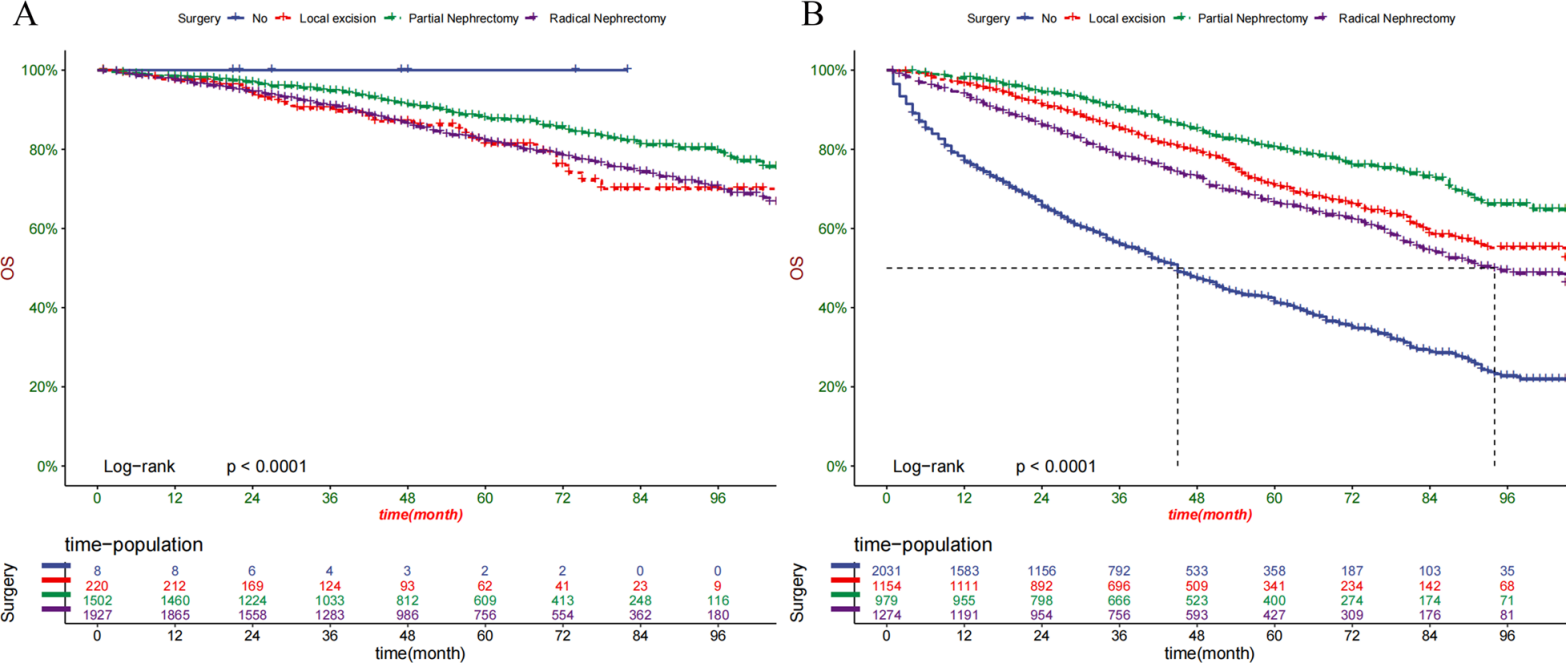
OS prediction of patients with different surgery in low- (**A**) and high-risk (**B**) group

DCAs showed that the clinical application value of nomograms was superior to that of the TNM stage system in the training cohort and the validation cohort (Fig. 5A–D). The DCA of externally validation suggested that the predictive model has good clinical value (Fig. 6A, B). We developed a risk stratification system based on the overall score of patients on a nomogram. Patients were divided into three groups: low-risk group (total score ≤ 62.3), high-risk group (total score > 62.3). Indeed, in the training and validation cohort, RCC patients in the high-risk group showed a shorter OS than patients in the low-risk group. The Kaplan–Meier curve shows that in all cohorts, the 1-, 3-, and 5-year OS rates of the low-risk group were 97.8%, 92.4%, and 84.5%, respectively; the high-risk group were 88.8%, 73.5%, and 60.7%, respectively (Fig. 7A, B). In addition, the impact of different surgical methods on the survival probability of patients in the low-, and high-risk groups was summarized. In the low-risk group, almost everyone has undergone surgery, patients with PN have the highest survival probability, followed by patients with RN and patients with local resection (Fig. 8A). In the high-risk group, most patients did not receive surgery or RN (Fig. 8B).

### Online application for OS prediction

Based on the nomogram, we have developed an easy-to-use online application to predict the OS of elderly RCC patients, which can be accessed at https://jinkuiwang.shinyapps.io/DynNomapp/. Enter the patient characteristics, you can immediately get the estimated survival probability. In a word, this online prediction tool is convenient to use in the clinic.

## Discussion

This study used a large number of clinical samples to establish a nomogram to predict the mid-term to long-term prognosis of elderly patients with early RCC based on the data from the US Surveillance, Epidemiology, and Final Outcome (SEER) program. We build the nomogram model and validate the performance of the model by screening clinically significant variables. In clinical practice, TNM stage is a routine method for doctors and researchers to evaluate tumor prognosis and select treatment strategies [25]. The nomogram survival prognosis prediction method has higher accuracy than the traditional TNM tumor stage system [26, 27]. Clinicians can predict the survival of patients based on the nomogram to help the elderly patients with early RCC formulate treatment strategies and answer patients’ consultations. As far as we know, there is no nomogram on the prognosis of elderly patients with early RCC. Therefore, it is necessary to establish a reliable prognostic model. We used the latest data to extract data from 11,976 elderly RCC patients from 2010 to 2018 from the SEER database. Consistent with previous studies, most patients are male, white, and married, and have undergone surgery [4].

RCC is the most common renal malignant tumor, accounting for about 85% of adult renal malignant tumors [28]. With the improvement of imaging technology, patients with small renal cell carcinoma have the opportunity to be detected early. Although the rate of new diagnoses in young patients is accelerating, elderly patients still have the highest overall risk of kidney cancer [12]. Overall, the 5-year relative survival rates of RCC were 81% (stage I) and 74% (stage II), but it dropped to 53% (stage III) and 10% (stage IV) [29, 30]. Therefore, it is of great significance to the care of the elderly patients with early RCC. Accurately predicting the outcome of elderly patients with early RCC can benefit from future treatment strategies and follow-up guidance.

Multivariable analysis found that age, histologic type, Fuhrman grade, T stage, surgery type, tumors number, tumor size, and marriage have a significant impact on the OS of RCC patients. It is easy to imagine that the increase of age leads to the decline of the patient's immune system, which will contribute to the deterioration of the tumor and reduce the survival time of the patient. Specifically, in this study, marriage is a factor that significantly affects OS. It may be that married patients get more emotional comfort and financial support from their family members, and thus have a better prognosis [31]. The effect of gender on patients may be related to hormone levels in the body [32]. There are more male RCC patients than females, and the prognosis is worse than that of females.

Fuhrman grade, another independent factor of OS in elderly patients with RCC. Consistent with previous studies, tumor grade is a significant prognostic risk indicator [16]. The nomogram model suggests that undifferentiated pathological types predict worse clinical outcomes. Pathological classification shows that compared with clear cell RCC, papillary and chromophobe RCC have a higher OS, because non-clear cell RCC tends to have a slower course of disease and much lower metastatic potential [33]. In our study, most patients have clear cell RCC, so surgical treatment has better OS than non-surgical treatment. In most patients, for early RCC in the elderly, monitoring is recommended to be limited to patients with substantial comorbidities, who are too weak to tolerate surgery. Depending on the T stage, the OS of elderly patients with RCC also differs, because RCC may show different growth depending on its size. Studies have shown that small tumors tend to grow at a relatively slow rate [34]. In our study, the results showed that there was significant difference in OS between T stages. The results of tumors number show that it has a significant impact on the OS rate of patients. It is not difficult to speculate that the number of tumors is negatively correlated with the prognosis of patients.

In this study, we found that for elderly patients with early RCC, PN can achieve the best OS, followed by local tumor excision, RN and non-surgical treatment. In the low-risk group, almost everyone has undergone surgery, although the difference between the various surgical methods is not obvious, the surgery seems to benefit the patient. In the medium-risk group, patients with PN have the highest survival probability. For medium-risk patients, the benefit of surgery is significantly higher than that of non-surgery. And it seems that PN can get a higher OS than RN in medium-risk patients. In high-risk patients, most patients have not undergone surgery, because these patients have a higher chance of comorbidities, and surgery may reduce the OS rate of patients.Although studies have suggested that age-related mortality from competitive causes may diminish the benefits of surgery for some elderly patients with early RCC [35]. Our results indicate that surgery in elderly patients with early RCC can improve the 1-, 3-, and 5-year OS. Indeed, the OS of patients undergoing PN was higher than that of patients undergoing RN, which is consistent with some previous observational studies [36–38], which may be related to the morbidity and mortality associated with chronic kidney disease. There are also studies indicate that the treatment for early stage renal cancer, especially T1a RCC, PN and RN have the equivalent treatment results, but the risk of subsequent chronic kidney disease is reduced [39–41]. In addition, local tumor excision also seems to be beneficial to the patients OS than non-surgery, even slightly better than RN. Due to local excision can prevent the progression of the tumor, compared with non-surgical treatment, it can benefit elderly patients with early RCC.

The parameters of the nomogram model constructed in this study include age, histologic type, Fuhrman grade, T stage, surgery type, tumors number, tumor size, and marriage, which can be easily collected in clinical practice. We used DCA to validate the accuracy and predictive ability of nomogram for elderly RCC patients. In short, nomogram had been proved to be able to accurately predict the OS of elderly RCC patients at 1-, 3- and 5-year, and has good clinical application potential. The risk stratification of our nomogram helps to identify high-risk groups, so as to provide accurate surgical intervention and monitoring for high-risk groups. In addition, we used the data of patients in 2018 as external validation. Short-term survival prediction showed that our prediction model has good accuracy and reliability. For elderly patients with early-stage renal cell carcinoma, 1-, 3-, 5-year survival rates can be obtained only by entering corresponding clinicopathological features according to the nomogram.

Our research also has some limitations. Because SEER does not collect data on the comorbidity of patients, the comorbidity of patients must increase with age, which affects the survival of patients. However, we have carried out a detailed stratification of the patient's age, which largely avoids the deviation of OS caused by aging and comorbidity. Also, since the data includes the years 2010–2018, with the growth of imaging technology and the improvement of surgery, the OS of patients may be different. However, we stratified according to the year of diagnosis and did not see obvious OS differences, indicating that our research is still applicable to the contemporary era. In addition, the SEER database is limited, we did not include some potentially important indicators, such as family history of hypertension, BMI, smoking and drinking, and genetic markers [42, 43]. Finally, although we have conducted external validation, large sample prospective clinical trials still needs to further validate the established prediction model.

## Conclusions

In summary, we have established a new monogram to predict the prognosis of elderly patients with early RCC based on a large population cohort in the SEER database. This tool can help doctors and elderly patients with early RCC to predict prognosis and formulate treatment and follow-up strategies.

## Data Availability

The data analyzed in this study is available at https://seer.Cancer.gov/.
